# Genome sequencing and genomic characterization of a tigecycline-resistant *Klebsiella pneumoniae* strain isolated from the bile samples of a cholangiocarcinoma patient

**DOI:** 10.1186/s13099-014-0040-2

**Published:** 2014-09-27

**Authors:** Beiwen Zheng, Ang Li, Xiawei Jiang, Xinjun Hu, Jian Yao, Lina Zhao, Jinru Ji, Min Ye, Yonghong Xiao, Lanjuan Li

**Affiliations:** State Key Laboratory for Diagnosis and Treatment of Infectious Diseases, The First Affiliated Hospital, School of Medicine, Zhejiang University, Hangzhou, 310003 China; Collaborative Innovation Center for Diagnosis and Treatment of Infectious Diseases, Zhejiang University, Hangzhou, 310003 China

**Keywords:** Tigecycline-resistant, *Klebsiella pneumoniae*, Cholangiocarcinoma, Next-generation sequencing, Comparative genomics

## Abstract

**Background:**

The relationship between *Klebsiella pneumoniae* and nosocomial and community-acquired infections is well known, and *K. pneumoniae* resistance to most antibiotics is increasing worldwide. In contrast, tigecycline remains active against many bacterial strains, and serves as a last resort for treating multi-drug resistant bacterial infections. That tigecycline nonsusceptibility among *K. pneumoniae* isolates has been reported worldwide is worrying. However, the mechanisms of tigecycline resistance in *K. pneumoniae* are less well known. We report the genome sequence and genomic characterization of tigecycline-resistant *K. pneumoniae* strain 5422 isolated from the bile samples of a patient with cholangiocarcinoma.

**Results:**

We sequenced the *K. pneumoniae* strain 5422 genome using next-generation sequencing technologies. Sequence data assembly revealed a 5,432,440-bp draft genome and 57.1% G + C content, which contained 5397 coding sequences. The genome has extensive similarity to other sequenced *K. pneumoniae* genomes, but also has several resistance-nodulation-cell division (RND) efflux pump genes that may be related to tigecycline resistance.

**Conclusions:**

*K. pneumoniae* strain 5422 is resistant to multiple antibiotics. The genome sequence of the isolate and comparative analysis with other *K. pneumoniae* strains presented in this paper are important for better understanding of *K. pneumoniae* multi-drug resistance. The RND efflux pump genes identified in the genome indicate the presence of an antibiotic resistance mechanism prior to antibiotics overuse. The availability of the genome sequence forms the basis for further comparative analyses and studies addressing the evolution of the *K. pneumoniae* drug resistance mechanism and the *K. pneumoniae* transcriptome.

## Background

*Klebsiella pneumoniae* is a Gram-negative opportunistic pathogen from the family *Enterobacteriaceae*. The increasing resistance detected in clinical isolates has become a matter of significant concern, and *K. pneumoniae* is an ESKAPE (*Enterococcus faecium*, *Staphylococcus aureus*, *K. pneumoniae*, *Acinetobacter baumannii*, *Pseudomonas aeruginosa*, and *Enterobacter* species) pathogen [1,2]. Infections caused by *K. pneumoniae* have been identified worldwide. It is worth noting that it is a major cause of nosocomial and community-acquired infections [3,4]. Moreover, a distinct invasive syndrome that causes liver abscesses has been detected in increasing numbers in Asia in the past two decades, and this syndrome is emerging as a global disease [5,6]. Furthermore, *K. pneumoniae* harboring extended-spectrum β-lactamases, and more recently, carbapenemase, which confers resistance to multiple antibiotics, has become a significant clinical concern worldwide [7,8]. Mortality among patients infected with extremely resistant *K. pneumoniae* is high, perhaps due to the limited therapeutic options remaining [9].

Tigecycline, a novel class of glycylcyclines, is a minocycline derivative synthesized to overcome the major tetracycline resistance mechanisms and to extend its spectrum of activity to multidrug-resistant (MDR) bacteria [10]. Tigecycline has enhanced antimicrobial activity compared to tetracycline, and can overcome efflux pump systems and ribosome protection mechanisms, retaining activity against a broad range of both Gram-positive and Gram-negative bacteria [11-13]. *K. pneumoniae* resistance to most antibiotics is increasing globally. Nevertheless, tigecycline remains active against many bacterial strains, and serves as last resort for treating MDR bacterial infections [14]. However, tigecycline resistance has emerged recently and been widely reported in *Enterobacteriaceae* isolates. It is worth noting that tigecycline non-susceptibility among *K. pneumoniae* isolates has been reported from different continents and ranges between 0% and 50% [15-17].

Previous studies have suggested that tigecycline resistance in *Enterobacteriaceae* is attributed to resistance-nodulation-cell division (RND)-type efflux pumps and transcriptional regulators of the efflux pump systems [15]. However, reports on the tigecycline resistance mechanisms in *K. pneumoniae* are rare. We hypothesized that a wide range of genes is involved in tigecycline resistance and contribute to decreased tigecycline susceptibility in *K. pneumoniae*, and performed whole-genome sequencing (WGS) to investigate this. We report the genome sequence of the tigecycline-resistant *K. pneumoniae* strain 5422 isolated from the bile samples of a patient with cholangiocarcinoma.

## Methods

### Strain information and growth conditions

Previously, we isolated strain 5422 from a bile sample obtained from a 54-year-old woman with bile duct cancer on February 25, 2012. After enrichment in Mueller-Hinton broth, the strain was identified as *K. pneumoniae* following the combination of its 16S rRNA gene sequencing and biochemical reaction results (VITEK 2 compact, bioMérieux, France). The strain exhibited high resistance to ciprofloxacin, cefotaxime, cefoxitin, ampicillin/sulbactam, sulfamethoxazole, tigecycline, tetracycline, and piperacillin, and was susceptible to imipenem, meropenem, and gentamicin. Multi-locus sequence typing revealed that it belonged to ST37 (unpublished data). The strain we reported here is available in the State Key Laboratory for Diagonosis and Treatment of Infectious Diseases, Zhejiang University.

### Genomic DNA extraction

Late log-phase cells were harvested and lysed with EDTA, lysozyme, and detergent treatment, followed by proteinase K and RNase digestion. Genomic DNA was extracted using a DNeasy Blood & Tissue Kit (Qiagen, Germany) according to the manufacturer’s recommended protocol. Genomic DNA yield, purity, and concentration was evaluated using 0.7% agarose gel electrophoresis with λ-*Hin*d III digest DNA Marker and measured using a NanoDrop 1000 Spectrophotometer (Thermo Fisher Scientific, USA). The genomic DNA was stored at-20°C.

### Genome sequencing and annotation

Whole-genome shotgun sequencing of *K. pneumoniae* strain 5422 was performed using a standard run of IlluminaHiSeq2000 sequencing by generating paired-end libraries (500-bp insert size) with a 2 × 100 pair-end sequencing strategy according to the manufacturer’s instructions. Clean reads were assembled into scaffolds using Velvet version 1.2.07 [18], and then we used PAGIT (Post-Assembly Genome Improvement Toolkit) [19] to extend the initial contiguous sequences (contigs) and correct sequencing errors. We identified tRNAs and rRNAs using tRNAscan-SE [20] and RNAmmer [21], respectively. Open reading frames (ORFs) were identified using Glimmer version 3.0 [22]. The genome was annotated using the RAST (Rapid Annotation using Subsystem Technology) server [23]. The classification of some predicted genes and pathways was analyzed using the COGs (Clusters of Orthologous Groups of proteins) [24] and KEGG (Kyoto Encyclopedia of Genes and Genomes) [25] databases. Stretches of amino acids containing the efflux pump genes were searched using BLAST (Basic Local Alignment Search Tool, http://blast.ncbi.nlm.nih.gov/Blast.cgi); protein-coding sequences were further BLAST-searched against the Antibiotic Resistance Database (ARDB) [26]. To find genes with hypothetical or putative functions, we aligned genes against the National Center for Biotechnology Information (NCBI) nucleotide sequence database (downloaded September 20, 2013) using NCBI BLASTn: we accepted only hits with identity of ≥ 0.95, coverage ≥ 0.9, and putative or hypothetical reference gene annotation.

### Initial comparative genomic and phylogenetic analysis

For comparative analysis, we downloaded the reference genome sequences of the closest genetic relatives of *K. pneumoniae* strain 5422 and representative strains from the NCBI website: *K. pneumoniae* LCT-KP182 (ATRN00000000), *K. pneumoniae* LCT-KP289 (ATRO00000000), *K. pneumoniae* subsp. *pneumoniae* LZ (AJVY00000000), *K. pneumoniae* ATCC BAA-2146 (AOCV00000000), *K. pneumoniae* 12 3578 (PRJNA199972), *K. pneumoniae* NB60 (AZAP00000000), *K. pneumoniae* subsp. *pneumoniae* 1084 (CP003785), *K. pneumoniae* subsp. *pneumoniae* NTUH-K2044 (AP006725), *K. pneumoniae* subsp. *pneumoniae* WGLW5 (AMLO00000000), *K. pneumoniae* ATCC 25955 (AQQH00000000), and *K. pneumoniae* G5-2 (AQQI00000000). Whole-genome alignments, single-nucleotide polymorphism (SNP) identification, and phylogenetic tree construction were performed using snpTree version 1.1, a server for online automatic SNP analysis of assembled genomes (http://cge.cbs.dtu.dk/services/snpTree-1.1/) [27].

## Quality assurance

The 16S rDNA gene from the draft genome was used to check for contamination. Further VITEK biochemical identification data confirmed that the strain 5422 belonged to *K. pneumoniae*. Bioinformatics assessment of potential contamination of the genomic library by allochthonous microorganisms was achieved using the BLAST non-redundant database.

## Initial findings

### Genome characteristics and phylogenetic analysis

**Figure 1:**
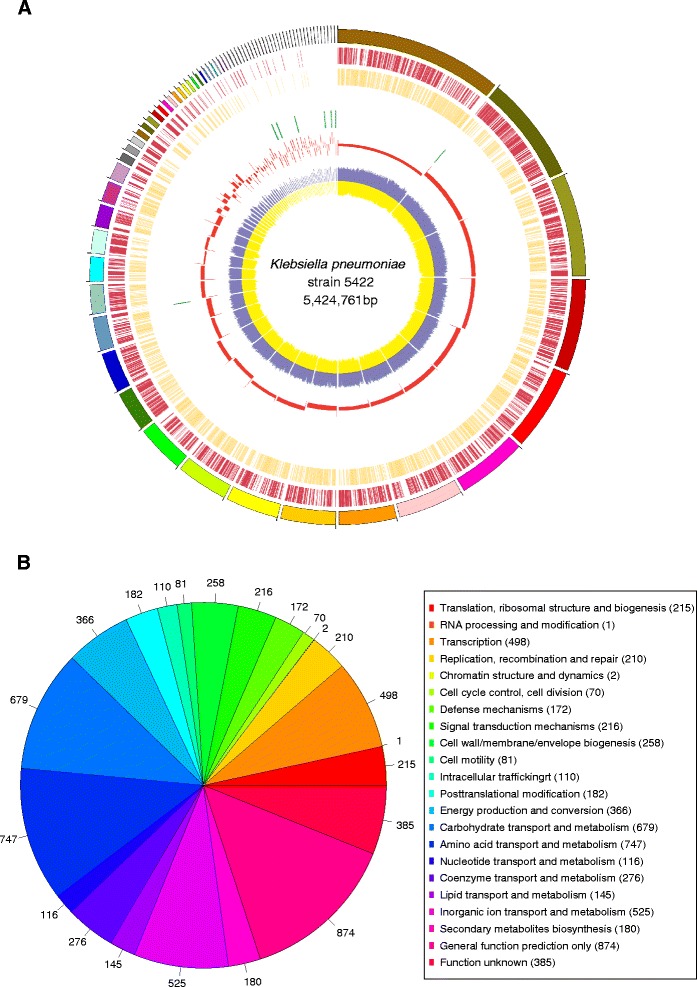
**Genome map and phylogenetic analysis. A**. Circular map of the genome generated using Circos. Circles from outside to inside: 1, contigs were arrange in clockwise direction from large to small; 2, CDS on forward strand; 3, CDS on reverse strand; 4, tRNA genes; 5, rRNA genes; 6, GC-skew (window size of 10 kb); and 7, blue indicates C content and yellow indicates G content (step size 500 bp). **B**. COGs distribution of *K. pneumoniae* strain 5422.

Filtered 520.8 M clean reads were assembled into scaffolds, and corresponding 99-fold coverage of the genome was generated. The draft genome sequence of *K. pneumoniae* strain 5422 was 5,432,440 bp in size and had a G + C content of 57.1% in 133 contigs, with N50 spanning 105,586 bp. Figure 1A depicts the overall genome profile. Annotation of this assembly identified 5,397 coding sequences (CDSs), 65 tRNAs (excluding 0 pseudo tRNAs), and incomplete rRNA operons (three small subunit rRNAs, four large subunit rRNAs). We assigned putative function or hypothetical proteins to 1,478 protein-coding genes. We categorized 4,218 genes into COGs functional groups (including putative or hypothetical genes, Figure 1B). For COGs distribution, R (general function prediction only; 874 ORFs), E (amino acid transport and metabolism; 747 ORFs), G (carbohydrate metabolism and transport; 679 ORFs), and P (inorganic ion transport and metabolism; 525 ORFs) were abundant categories (>10% of total COGs matched counts).

**Figure 2:**
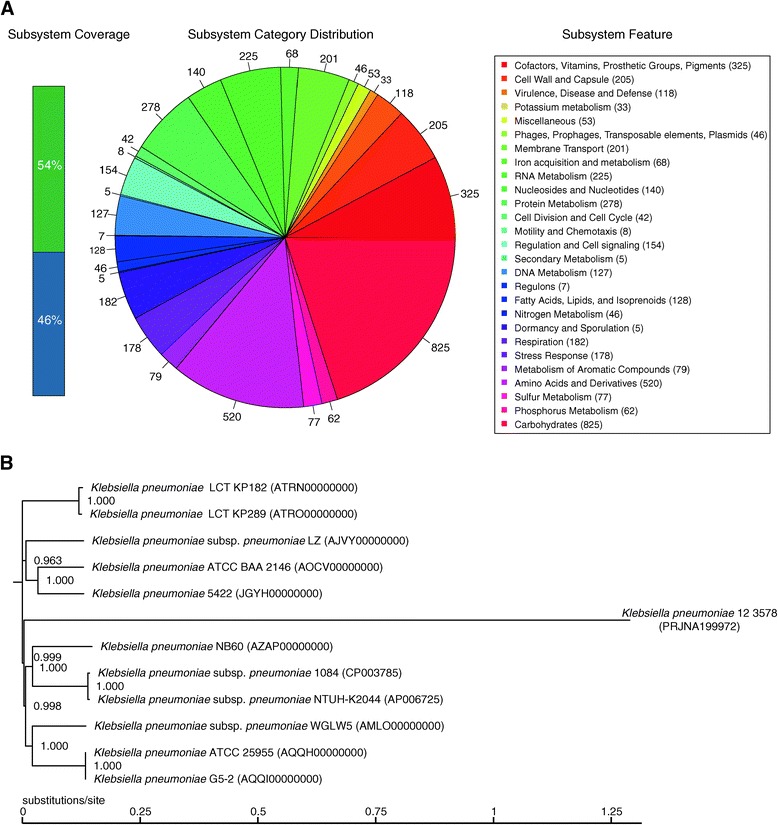
**Subsystem distribution and phylogenetic analysis. A**. Distribution of genes assigned to SEED subsystems (based on the RAST annotation server). **B**. Phylogenetic relationships (based on WGS and SNPs) of 12 *K. pneumoniae* strains and their genomic distance analysis. The snpTree server output used assembled genomes as input data.

Figure 2A illustrates the subsystem distribution and general information on the potential functional distribution of *K. pneumoniae* strain 5422. Genes responsible for carbohydrates (825 ORFs); amino acids and derivatives (520 ORFs); and cofactors, vitamins, prosthetic groups, and pigments (325 ORFs) were abundant among the SEED subsystem categories. Based on the raw reads and assembled genomes from published *K. pneumoniae* WGS data sets, we conducted phylogenetic analysis on tree topology and the SNP positions of the reference genome to identify the most closely related organism. The phylogenetic tree based on whole-genome SNPs showed that the closest ancestor to *K. pneumoniae* strain 5422 was *K. pneumoniae* ATCC BAA-2146 (Figure 2B), which is the first US isolate found to encode New Delhi metallo-β-lactamase 1 (NDM-1), eight β-lactamases, and 15 additional antibiotic-resistance enzymes [28,29].

### RND efflux pumps in K. pneumoniae strain 5422

The RND family members are important mediators of MDR in Gram-negative bacteria. The AcrAB-TolC system in *Escherichia coli* and the MexAB-OprM complexes in *P. aeruginosa* are extremely well characterized, and the three-dimensional structures of various components have been resolved [30]. In the genome of *K. pneumoniae* strain 5422, 16 genes were indicated as probable efflux pumps or translational regulators based on their sequence similarity to known RND efflux pump genes (Table 1).

**Table 1:** **Summary of CDSs annotated to RND efflux pump genes**

**Aligned protein**	**Query sequence length**	**Coverage (%)**	**Hit length**	**Identity (%)**	**Description**
AcrA	240	64.17	146	60.08	Probable RND efflux membrane fusion protein
OqxB	217	20.66	141	64.98	Probable RND efflux system inner membrane transporter CmeB
OqxB	181	18.53	110	60.77	Probable RND multi-drug efflux transporter
OqxB	160	15.27	105	65.62	Probable RND efflux system inner membrane transporter CmeB
AcrA	397	100	397	100	Membrane fusion protein of RND family multi-drug efflux pump
OqxR	483	100	480	99.38	Transcriptional regulator
OqxB	1050	100	1050	100	RND multi-drug efflux transporter
OqxA	391	100	391	100	RND multi-drug efflux transporter
RarA	369	100	366	99.19	Bacterial regulatory helix-turn-helix proteins, AraC family
OqxB	161	15.33	101	62.73	Probable RND multi-drug efflux transporter
AcrA	328	83.89	248	75.61	Probable RND efflux system membrane fusion protein CmeA
OqxB	217	20.67	143	65.9	Probable RND efflux system inner membrane transporter CmeB
TolC	1520	100	1474	96.97	Outer membrane efflux protein
OqxB	326	31.05	200	61.35	Probable RND efflux system inner membrane transporter CmeB

## Future directions

The rapid progress of WGS has permitted detailed investigation of genetic differences between bacterial isolates with different phenotypic characteristics. Whole-genome studies of *K. pneumoniae* have mainly focused on comparing either distinct antibiotic-susceptible and MDR strains or related isolates from different patients. Therefore, large-scale genomic sequencing and comparative genome analysis of tigecycline-resistant*,* tigecycline non-susceptible, and tigecycline-susceptible clinical isolates will identify the differences in the genomic content of this species and yield evolutionary information on the development of tigecycline resistance through mutations. Moreover, further studies involving extensive high-throughput mRNA sequencing (RNA-Seq) experiments to significantly improve annotation and to provide exceptionally robust analysis of RNA expression under selective antibiotic pressure are warranted.

### Ethics approval

This research was approved by the Research Ethics Committee of the First Affiliated Hospital, School of Medicine, Zhejiang University, and informed consent was obtained from the patient.

## Availability of supporting data

This Whole Genome Shotgun project has been deposited at DDBJ/EMBL/GenBank under the accession JGYH00000000. The version described in this paper is version JGYH01000000.
